# Combined Interventions to Reduce Burnout Complaints and Promote Return to Work: A Systematic Review of Effectiveness and Mediators of Change

**DOI:** 10.3390/ijerph17010055

**Published:** 2019-12-19

**Authors:** Roald Pijpker, Lenneke Vaandrager, Esther J. Veen, Maria A. Koelen

**Affiliations:** Department of Social Sciences, Wageningen University & Research, 6700 EW Wageningen, The Netherlands; Lenneke.Vaandrager@wur.nl (L.V.); Esther.Veen@wur.nl (E.J.V.); Maria.Koelen@wur.nl (M.A.K.)

**Keywords:** burnout, combined interventions, mediators of change, occupational health, PRISMA, resources, rehabilitation, return to work, systematic review, workforce

## Abstract

Burnout has adverse effects on the health and work-related outcomes of employees. Nevertheless, little is known about effective ways of reducing burnout complaints and facilitating full return to work, which defines rehabilitation. This study consists of a systematic review of the effects of combined interventions (i.e., both person-directed and organization-directed). It also includes the identification and description of mediators of change, thereby explaining how combined interventions do or do not work. Seven electronic databases were searched for English peer-reviewed publications: the Psychology and Behavioral Sciences Collection; PsycARTICLES; Web of Science; Scopus; SocINDEX; PubMed; and PsycINFO, using various combinations of search terms (e.g., burnout AND intervention). Out of 4110 abstracts published before 29 September, 2019, 10 studies (reporting the effects of nine combined interventions) fulfilled the inclusion criteria, which were defined using PICOS criteria (participants, interventions, comparators, outcomes and study design). Although the risk of bias of the included studies is high, all combined interventions were effective in facilitating rehabilitation. Results suggest that involving employees in decision-making and enhance their job control and social support, while eliminating stressors, explain the effectiveness of the intentions. With caution, workplace health promotion practitioners are encouraged to use these findings to tackle burnout among employees.

## 1. Introduction

In the countries of the Organization for Economic Co-operation and Development (OECD), work-related stress is the leading cause of absenteeism [1], with significant financial consequences for society [2]. The best-known occupational syndrome—burnout—has adverse effects on the health and wellbeing of employees (e.g., increasing physical illness [3]), in addition to affecting their attitudes at work (e.g., decreasing organizational involvement [4,5]). For instance, burnout has shown to be an important correlate of musculoskeletal disorders (e.g., chronic back pain), which, in turn, are associated with a further increase in burnout complaints, daily productivity loss and form a major cause of occupational leave and prolonged recovery time [6,7]. Moreover, burnout and its adverse effects on the health and wellbeing of the workforce, is associated with high rates of sick leave and replacement costs [8,9]. It is therefore of the utmost importance to tackle burnout, both for employee health and wellbeing and for organizational development and performance.

Burnout is predominantly described as an outcome of “a prolonged response to chronic emotional and interpersonal stressors on the job, defined by the three dimensions of exhaustion, cynicism and professional efficacy” [10] (p. 397). Emotional exhaustion refers to a feeling of being depleted and overextended by one’s emotional and physical resources. Cynicism and depersonalization refer to a detached response to various aspects of the job. Reduced efficacy and accomplishment refer to a sense of incompetence and lack of productivity at work [11]. Burnout complaints can occur in employees who are currently still working. Over time, however, burnout can lead employees to take sick leave and become unable to work [12].

According to several theories, burnout develops in a non-linear manner [13]. Models that are well-supported by empirical evidence include the Job Demand-Control Model [14], Conservation of Resources theory [15] and the Job Demands-Resources Model [16]. These models emphasize that the development of burnout is fostered through a complex interplay between factors within employees (e.g., low self-esteem) and factors within the organizational context (e.g., work overload). Based on these theories, interventions should target both employees and their working contexts, in order to facilitate rehabilitation (i.e., reducing burnout complaints and promoting full return to work (RTW)) [17]. Examples of person-directed interventions include psychotherapy and mindfulness sessions. Examples of organization-directed interventions include changing working schedules and team building.

### 1.1. Scientific Gap

To date, most systematic reviews and meta-analyses have focused separately on either person-directed or organization-directed interventions, both of which have proven suboptimal in facilitating rehabilitation [18]. Their lack of effectiveness has consequently been attributed to the single-level approach (either person-directed or organization-directed interventions) [18]. There is thus a need to synthesize the effectiveness of existing combined interventions (both person-directed and organization-directed). Although existing theories suggest that combined interventions could be effective in facilitating rehabilitation, it would also be interesting to examine why and how interventions do or do not work. This has yet to be sufficiently understood [18,19,20]. In studies on interventions aimed at reducing stress-related complaints in general (and not specifically burnout), job control has been found to mediate changes in such complaints [21]. It would therefore be worthwhile to explore the role of possible mediators of change in combined interventions.

### 1.2. Study Objective

Based on studies with experimental designs, the present study aims to assess the effectiveness of combined (both person- and organization-directed) interventions for employees with burnout complaints (currently either working or not working) on facilitating rehabilitation. Complementary, this review aims to identify and describe mediators of change that could explain how combined interventions do or do not work.

## 2. Methods 

The systematic review was structured according to the Preferred Reporting Items for Systematic Reviews and Meta-Analyses (PRISMA) guidelines [22]. More specifically, we used the PRISMA checklist to guide the design and reporting of the systematic review (supplementary materials, Table S1). Since we did not aim to conduct a meta-analysis of the combined interventions, not all elements of this checklist were relevant for this review (e.g., statistical measures of consistency).

### 2.1. Inclusion Criteria 

Five inclusion criteria were applied to the identified studies, based on the PICOS criteria (participants, interventions, comparators, outcomes and study design). First, to reduce heterogeneity between studies, those focusing on employees were included, while those focusing on students [23], athletes [24] and volunteers [25] were excluded. Second, combined interventions (both person-directed and organization-directed) were included. Third, we did not define a comparison exposure, which means that experimental studies that did not include a control group were included. Fourth, studies using the Maslach Burnout Inventory (MBI) to assess burnout were included, as the MBI is regarded as the gold standard for measuring burnout [26], thereby enhancing comparability between studies. With respect to return to work (RTW), all operationalizations were included. Fifth, randomized controlled trials, quasi-experimental and pre-test/post-test study designs were included, as these designs provide more robust evidence than do cross-sectional or other non-experimental designs [27]. Finally, only studies published in English between 1970 and 29 September 2019 were included.

### 2.2. Data Sources and Search Terms

Seven electronic databases were searched for peer-reviewed publications: the Psychology and Behavioral Sciences Collection; PsycARTICLES; Web of Science (all databases); Scopus; SocINDEX; PubMed; and PsycINFO. Search terms were based on the three dimensions of burnout—emotional exhaustion, depersonalization *or* cynicism, personal accomplishment *or* professional efficacy, Maslach Burnout Inventory *or* MBI—and combined with *and* “intervention”. To ensure substantial breadth and depth in the electronic databases, the search strategy was pilot tested before the search was conducted. The first author conducted the electronic search. 

### 2.3. Search Strategy

**Figure 1:**
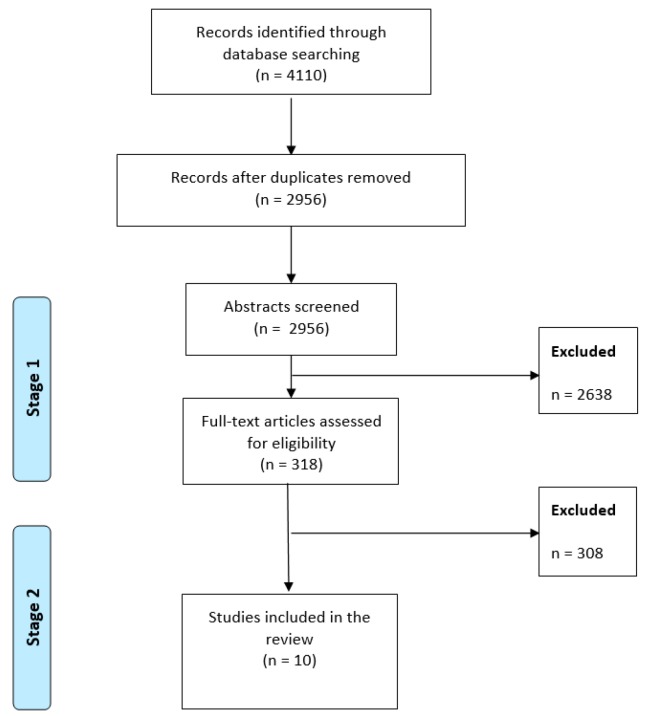
Process of study selection.

The database search yielded 4110 hits, including a large number of duplicates (n = 1154). The subsequent search strategy consisted of two stages (See Figure 1). In the first stage, titles and abstracts were screened against the inclusion criteria and abstracts deviating from them were excluded (n = 2638). Where the reviewer was uncertain, the abstract was moved onto the next stage for a full-text review. In the second stage, full-text articles were screened (n = 318) against the inclusion criteria. Studies that did not meet the inclusion criteria (e.g., non-combined interventions) were excluded (n = 308). The first three authors were involved in the full-text screening stage and, in case of uncertainty, the fourth authors acted as tie-breakers for the inclusion or exclusion of the remaining articles. The reference lists of the included articles were screened to identify any additional relevant studies. These lists did not reveal any additional studies and 10 studies were ultimately included in the review. 

### 2.4. Data Extraction and Quality Assessment

The data extraction phase consisted of two steps. In the first step, studies were described according to the following characteristics: author(s) and country; setting and design; study aim and outcomes; participants; controls; theoretical framework; interventions; mediators of change; duration and frequency; pre-test, post-test and follow-up; and results. In the second step, statistically significant effects of the combined interventions on the reduction of burnout complaints and the promotion of RTW were described, as were the theoretical assumptions and mediators of change. All researchers were actively involved in defining how the data should be extracted and described in an iterative process.

The theoretical assumptions underlying the combined interventions were described in order to enhance insight into why the interventions did or did not work. For combined interventions that were not built on any specific theory, it was deemed appropriate to describe the general assumptions made by the authors (if described). With regard to mediators of change, in addition to reporting those mediators that were explicitly measured and evaluated concerning change in the outcome variables, we also described mediators of change that were identified in the theoretical (or other) assumptions underlying the combined interventions.

Since the overarching aim of this review is to assess the effectiveness of the combined interventions for the rehabilitation of employees with burnout, it is important to assess the risk of bias of the included studies to determine the extent to which the reported effects can be attributed to the interventions and not to a lack of methodological rigor. To assess this risk of bias, we used the Quality Assessment Tool for Quantitative Studies, which is specifically developed by the Effective Public Health Practice Project for this critical step [28]. The tool can be applied in any public health topic area and has been evaluated on its validity and reliability, which proved to be strong [28,29]. The tool defines six components to assess the risk of bias: selection bias (e.g., do the study participant represent the target population?), study design (e.g., was a randomized controlled trial design used?), confounders (e.g., how did the authors deal with possible differences between experimental and control groups?), blinding (e.g., were the study participants aware of the research question?), data collection methods (e.g., were the measurements instruments reliable and valid?), withdrawals and dropouts (e.g., were withdrawals and dropouts reported?) [28]. The first author conducted the assessment and nine studies showed a “high” risk of bias and one study was assessed as having a “moderate” risk of bias. Studies were not excluded based on this assessment; however, the results should be interpreted with caution. To ensure transparency, the assessment scores can be found in the Supplementary materials, Table S2. 

## 3. Results

### 3.1. Description of the Studies

Descriptive information regarding the 10 studies in this review is presented in Table 1.

#### 3.1.1. Countries, Settings and Research Designs

The articles were based on studies conducted in eight countries: USA (n = 2), Sweden (n = 2), the Netherlands (n = 2), Norway (n = 1), Finland (n = 1), Hong Kong (n = 1) and England (n = 1). The settings were highly heterogeneous, ranging from self-employed individuals to white-collar workers and healthcare workers. The research designs also varied, ranging from a controlled clinical trial to pre-test/post-test designs. This was also the case for the time between pre-tests and follow-up measurements, which ranged from four months (Study 5) to 30 months (Studies 1 and 2).

#### 3.1.2. Measures of Burnout and RTW

Most of the studies concerned interventions aimed at reducing burnout complaints (Studies 3, 4, 5, 6, 7, 8 and 9), although three aimed to promote full RTW (Studies1, 2 and 10). Multiple versions of the MBI were used across the studies, including the MBI—General Survey (measuring exhaustion, cynicism and professional efficacy) and the MBI—Human Survey Index (measuring exhaustion, cynicism and personal accomplishment). Studies 1, 2 and 10 measured RTW, which was operationalized as the mean number of days to partial and full RTW (Study 10) and the sick leave percentage (Studies 1 and 2).

#### 3.1.3. Combined Interventions, Theories and Mediators of Change

The combined interventions were all different in terms of content. More specifically, none of the studies evaluated the same person-directed and organization-directed interventions. The duration and frequency of the interventions also differed sharply across the studies, depending on the activities on which the interventions were based. For example, Study 9 was based on three sessions of three hours each, while Study 3 was based on six monthly sessions of four hours each. Multiple theoretical frameworks were identified, with most focused on job-person mismatch (Studies 1, 2, 4, 5 and 6). Study 8 used the Demand-Control-Support Model and Studies 3, 7, 9 and 10 did not report any theoretical framework.

Abbreviations: CCT = Controlled Clinical Trial; MBI = Maslach Burnout Inventory; MBI–GS = Maslach Burnout Inventory–General Survey; MBI–HSS = Maslach Burnout Inventory–Human Services Survey; MBI–NL = Maslach Burnout Inventory–Netherlands; RTW = Return to Work; EX = Exhaustion; CY = Cynicism; DP = Depersonalization; PA = Personal Accomplishment; PE = Professional Efficacy.

### 3.2. Effectitivness of the Combined Interventions 

#### 3.2.1. Studies 1 and 2

• Effectiveness

A controlled clinical trial design was conducted among Swedish employees on sick leave due to burnout. The authors conclude that, after 18 months, 89% of the employees in the experimental group had RTW to a certain extent, whereas only 73% employees in the control group had RTW. The effect of the combined intervention remained stable after 30 months, where 82.4% of the employees where back at work, which was still a higher percentage compared to the control group (77.9%).

• Underlying Principles

The combined intervention was based on a convergence-dialogue meeting (CDM), which was intended to cultivate a dialogue between the employee and the supervisor to identify opportunities in order to facilitate RTW. 

• The combined Intervention 

To facilitate RTW, an outline of the employee’s perspective was compiled according to multiple sources (e.g., questionnaire replies, the course of events leading to burnout, the employee’s own views of changes required for RTW). Based on this outline, the supervisor was interviewed at the workplace, in addition to outlining the perceived causes of the employee’s absenteeism and the changes required in order to facilitate RTW. In general, the intervention focused on solutions and changes aimed at the identification of converging perspectives and goals between employees and supervisors.

The CDM started by highlighting the agreements and disagreements between the supervisor and the employee with regard to the causes of the sick leave and the improvements required in order to facilitate RTW. Each session lasted for about 1.5 hours, resulting in agreements concerning short term and long-term goals and solutions. Thereafter, the employees were invited to a seminar, along with 4–6 other employees who had participated in the intervention. The seminar consisted of discussions and lectures on the topic of work-related (and other) stress. These discussions and lectures were also arranged separately for the supervisors involved. For the employees, the seminar aimed to help them reflect on how they could prevent a similar occurrence of sick leave in the future. For the supervisors, the focus was on how to prevent sick leave related to work stress among their employees.

• Mediators of Change

The expectation that facilitating partial RTW would predict full RTW was not supported by empirical evidence. The principles underlying the combined intervention (e.g., an actual change in the work environment) were not evaluated.

#### 3.2.2. Study 3

• Effectiveness

A quasi-experimental study design was employed to investigate Dutch oncology staff with a risk of developing burnout. The authors conclude that, compared to the two control wards, the combined intervention resulted in significantly less exhaustion after both 6 and 12 months and in less depersonalization after six months.

• Underlying Principles

No specific theoretical framework was reported. Instead, the combined intervention was inspired by a participatory approach, in which the interventions were context-specific and based on an accurate assessment of both individual and organizational factors, rather than relying on pre-packaged, context-independent programs based on a uniform and theory-based approach. The combined intervention was developed in collaboration with external counselors and it combined a support group with the participatory approach. Before the intervention, intake interviews were held with the managers of the ward (e.g., discussing possible intervention effects), in order to enhance their motivation to implement the interventions.

• The Combined Intervention 

The combined intervention consisted of six monthly meetings of three hours each, which were supervised by both of the team counselors. The first session started with education on job stress (although no details were provided) and the results concerning the employees’ work situations (e.g., workload, emotional demands, job control, social support, participation in decision-making), as measured at T1, were fed back to the employees. During this first meeting, the participants selected a number of stressors to be addressed (e.g., lack of social support). Each of the remaining meetings consisted of two parts: education and action. The educational part focused on the emergence and persistence unwanted behavior (Meeting 2); feedback and communication (Meeting 3); creating a social support network (Meeting 4); and balancing job-related investments and outcomes (Meeting 5). The action part focused on enhancing the abilities of the workforce to cope with stressors effectively. Outcomes of these sessions included restructuring the weekly work meetings in order to allow more staff participation in decision-making.

• Mediators of Change

The authors conclude that the combined intervention significantly increased participation in decision-making, which subsequently led to a decrease in exhaustion. Similar results were reported for social support and job control: increases in both social support and job control were significantly related to simultaneous reductions in exhaustion and depersonalization. Even though participants in the combined intervention reported fewer burnout complaints than did those in the control group, both exhaustion and depersonalization had increased after one year. This result can be explained by an increase in the perceived workload (although it is not clear what contributed to this increase). 

#### 3.2.3. Study 4

• Effectiveness

A quasi-experimental study design was employed to investigate construction-related professionals engaged in property development, consulting and contracting companies in Hong Kong. One year after the combined intervention, employees reported significantly fewer feelings of exhaustion and cynicism than they had before the intervention.

• Underlying Principles

The combined intervention was conducted from the perspective of job-person mismatch, using job-redesign to reduce mismatches (i.e., stressors) identified through a cross-sectional study measuring job-related variables among employees (i.e., working hours, quantitative workload, role conflict, control over work pace, satisfaction with supervisor).

• The combined Intervention

The person-directed interventions included in-house training courses to enhance the ability of employees to cope actively with stressors (e.g., improving time management skills). The organization-directed intervention included a change in working hours (one additional day off every two weeks), which was expected to reduce the incidence of long working hours. The authors did not describe how the combined intervention was implemented or by whom. 

• Mediators of Change

The mediators of change (possible stressors) were measured only in order to inform the development of the combined intervention. They were neither tested nor evaluated with regard to the change in burnout complaints reported by employees. 

#### 3.2.4. Study 5

• Effectiveness

A quasi-experimental study design was employed in Finland to investigate white-collar workers (e.g., staff from social services and health departments) who were currently on sick leave due to burnout. The authors conclude that the combined intervention produced a significant reduction in feelings of exhaustion after both four and eight months and in cynicism after four months. Although no changes occurred in the person-directed intervention (Control group 1), cynicism decreased significantly in the no-treatment group (Control group 2). 

• Underlying Principles

The theoretical framework was based on job-person mismatch. During the intervention process, participants collaborated with representatives from their workplaces and with the rehabilitation staff to reduce mismatches and improve their working environment.

• The combined Intervention

The combined intervention entailed one year, with two rehabilitation periods (12 and 5 days, respectively). Person-directed interventions involved activities including physiological and occupational therapy, which was intended to enhance the abilities of employees to cope with stress and to promote awareness of stress-evoking situations and how people react to stress. The organization-directed intervention included a link to the workplaces of the rehabilitation clients. More specifically, the employee’s supervisor, a member of the occupational health and safety organization, plus a representative from occupational health care were invited to the rehabilitation center for one day during each rehabilitation period. The inclusion of representatives from the workplace in the rehabilitation process was intended to involve the employer and to create an obligation for the employer to implement the actions agreed upon in order to remedy defects in the workplace.

In the first rehabilitation period, the workplace-related representatives collaborated with the participants and the rehabilitation team to identify ways to improve job conditions for the participants, based on a memorandum that the participants had prepared in advance of this meeting. The memorandum included issues that the participants considered essential to enhancing personal job-related wellbeing and health. In the second rehabilitation period, the same individuals met at the rehabilitation center again and discussed whether the agreed upon remedies had been implemented and whether any problems had been related to them. This was expected to reduce burnout complaints. The purpose of these two meetings was to increase the control that employees had over issues relating to their jobs and to improve their job conditions.

• Mediators of Change 

The intervention assumed that a change in burnout complaints (feelings of exhaustion) would be mediated by a decrease in time pressure at work. The changes in two other burnout symptoms (cynicism and reduced professional efficacy) were expected to be mediated, particularly by an increase in perceived job control. Theoretically, the change in these symptoms was also expected to be mediated by improvements in the workplace climate and satisfaction with the supervisor. The results support that the combined intervention significantly increased employee job control and that this increase resulted in lower levels of exhaustion and cynicism over the 12-month rehabilitation process. Workplace climate had only a minimal (non-significant) mediating influence on exhaustion and no effect on cynicism.

#### 3.2.5. Study 6

• Effectiveness

A two-group pre-test/post-test design was employed to investigate community healthcare staff caring for people with disabilities in two municipalities in Norway. In that country, responsibility for people with disabilities was transferred from the county to municipal level in 1991. The authors conclude that the combined intervention significantly reduced feelings of exhaustion after 10 months, whereas no changes in burnout complaints were observed in the control group. 

• Underlying Principles

The combined intervention was conducted from the perspective of job-person mismatch. Rather than trying to eliminate all stressors (i.e., “mismatches”), the intervention assumed that focusing on mismatches that, if resolved, could potentially generate and allow the implementation of concrete solutions that would be most effective in reducing burnout complaints. It was also argued that the involvement of multiple stakeholders (i.e., the manager, two senior leaders, two employees, researchers and the human resource manager—the working group) and the support of the supervisor were critical success factors for any intervention in the workplace. 

• The Combined Intervention

Employees discussed stressors and potential solutions and this resulted in priority lists concerning actions aimed at improving working conditions. Based on this list, the working group agreed upon a set of intervention strategies to be implemented at both the individual and organizational levels. For example, the person-directed interventions entailed a voluntary exercise program, in which the study participants were expected to exercise to improve fitness. Three organization-directed interventions were implemented: the introduction of performance appraisals, the re-organization of working schedules to promote larger positions (i.e., more working hours each week) and stability among the staff and the improvement of routines for new employees (e.g., better on the job training). Specific goals were formulated for each of the interventions. For example, the purpose of the exercise program was to improve the health and wellbeing of employees, thereby buffering the adverse effects of burnout. The organization-directed interventions were intended to provide feedback to both employees and supervisors, in addition to promoting job security, which is assumed to reduce burnout complaints.

• Mediators of Change

Neither the underlying principles (e.g., enabling employees to participate in decision-making) nor the goals of the intervention (e.g., promoting job security) were evaluated with regard to the change in burnout complaints. 

#### 3.2.6. Study 7

• Effectiveness

A one group pre-test/post-test design was employed to investigate general surgery residents working at the University of Arizona (USA). The authors conclude that the combined intervention significantly reduced feelings of exhaustion after 12 months. 

• Underlying Principles

Although the combined intervention was not based on any specific theoretical framework, multiple assumptions were described. The intervention assumed that enhancing the self-awareness and emotional intelligence of employees would teach them to respond effectively, rather than reacting to the stress inherent in their lives and environments, thereby reducing burnout complaints.

• The Combined Intervention

The person-directed interventions entailed activities aimed at improving the work-life balance of employees and promoting healthy diet and exercise (none of which were explained). One organization-directed intervention was implemented, which entailed a range of team building activities (none of which were explained). The overall aim of the person-directed and organization-directed activities was to improve the mental, physical and social health and wellbeing of employees and this was assumed to reduce burnout complaints. The employees were asked to evaluate the program based on certain predefined indicators (which were not based on the underlying principles). The results indicated that, in general, the employees perceived the program as positive. For example, 96% strongly agreed that the program created cohesiveness and a sense of community among the workforce. 

• Mediators of Change

None of the underlying principles (e.g., enhancing employee self-awareness) was evaluated with regard to the change in burnout complaints, nor were the goals of the intervention (e.g., improving their health) or the predefined outcomes (e.g., promoting a sense of community). 

#### 3.2.7. Study 8

• Effectiveness

A one group pre-test/post-test design was employed to investigate staff working in an alcohol ward in England. The authors conclude that the combined intervention led to a significant increase in personal accomplishment after one month. 

• Underlying Principles

The combined intervention was based on the Demand-Control Support Job Stress Model, with an emphasis on enhancing social support among the workforce in order to improve their ability to cope with stress. Social support was assumed to act as a buffer against the possible adverse health effects of excessive psychological demands or stressors. Employees were involved (although it was not clear how) in identifying stressors, which were then used as a foundation for developing the combined intervention (although it was not clear how). The stressors identified by the staff included group work, dealing with complex clients and client aggression. The practice of working with whole teams was assumed to have a positive effect on the entire team culture, in addition to introducing bottom-up working practices aimed at reducing stress (none of these aspects were explained).

• The Combined Intervention

Although the combined intervention focused on working with the whole team, elements in the training also addressed both individual and organizational issues. In practice, the combined intervention consisted of two days of training, with two weeks between the training days. The focus of the first day of the training was on “Managing stress at the individual, team and organizational level,” and the second day was devoted to understanding “the causes and consequences of aggression.” One aspect of the training consisted of identifying the common antecedents of episodes of violence from a comprehensive perspective, including client-related, environmental, team and organizational factors, although none of these factors were explained. The team members then received assistance in undertaking a comprehensive risk assessment (although it was not clear what was being assessed) and strategies for implementing interventions to address the risks (these were also not explained).

• Mediators of Change

None of the underlying principles (e.g., the role of social support or employee participation) was evaluated with regard to the change in burnout complaints. 

#### 3.2.8. Study 9

• Effectiveness

A one group retrospective pre-test/post-test design was employed in the USA to investigate staff representing multiple healthcare professions (e.g., nursing, pharmacy, housekeeping). The authors conclude that the combined intervention resulted in a significant decrease in exhaustion and a significant increase in professional accomplishment.

• Underlying Principles

No specific theoretical framework was mentioned, nor was any rationale underlying the combined intervention.

• The combined Intervention

The combined intervention consisted of three sessions (workshops) of three hours each, focusing on team building (positive human connections), communication skills, building self-esteem and stress management. Building self-esteem and stress management are person-directed interventions, while team building is an organization-directed intervention. For example, the team building exercises included the construction of straw towers in small groups, a values-clarification exercise and the preparation and performance of a musical number in which each person acted as an instrument. Examples of stress management techniques included breathing exercises, guided visualization and a shoulder massage with a co-worker. 

• Mediators of Change

Some mediators of change were subjected to qualitative exploration and quantitative description, expressing how often a particular theme was mentioned. For example, employees reported that the combined intervention resulted in better communication with co-workers (24%), a better working atmosphere (53%) and increased self-esteem (18%). However, none of these mediators was evaluated with regard to changes in burnout complaints reported by the employees.

#### 3.2.9. Study 10

• Effectiveness

A controlled clinical trial design was employed in the Netherlands to investigate self-employed individuals (i.e., business owners) who were currently on sick leave due to burnout. Participants in the combined intervention partially returned to work 17 and 30 days earlier than did their counterparts in the person-directed intervention and the control group. For full RTW, this difference was approximately 200 days. All of these differences were statistically significant. When controlling for gender, age, education and number of employees, however, the effect of the combined intervention was no longer significant for partial RTW, although it did persist for full RTW.

• Underlying Principles

No specific theoretical framework was reported. The intervention had a strong focus on graded activity. More specifically, it involved a process of gradual exposure, in which the participant’s activation was increased through small steps. Six labor experts participated in the study. All of these experts received training in a brief stress management intervention based on cognitive based therapy. The stress management part of the intervention consisted of psycho-education on work stress, the registration of symptoms and situations, relaxation, self-help books on rational emotive behavior therapy and assignments involving writing and time management. In addition, the labor experts advised the participants with regard to work processes and provided suggestions for reducing workload and job demands while increasing decision latitude. These components were intended to foster at least partial work resumption. 

• The combined Intervention 

The combined intervention consisted of five to six sessions of approximately one hour, twice a week. A person-directed (based on Cognitive Based Therapy) intervention was combined with an organization-directed intervention focusing on reducing stressors at work (e.g., reducing workload).

• Mediators of Change

None of the underlying assumptions (e.g., increasing the employees’ decision latitude) was either empirically tested or evaluated with regard to change in RTW. 

## 4. Discussion

### 4.1. Summary of Findings 

This study consisted of a systematic assessment of combined interventions with regard to their effectiveness, theoretical assumptions and mediators of change. Of the 4110 abstracts obtained in a literature search (published before September 29, 2019), 10 studies (reporting the effects of 9 combined interventions) fulfilled the predefined inclusion criteria. It should be emphasized that all 9 combined interventions were effective (at least to a certain extent) in facilitating rehabilitation. With regard to the reduction of burnout complaints, the combined interventions led to greater improvement in exhaustion and cynicism (or depersonalization), in both the short term (after 4 months) and the long-term (after 12 years), than in professional efficacy (or personal accomplishment). In terms of promoting RTW, the combined interventions showed long-term effects on the promotion of full RTW.

Surprisingly, very few of the studies devoted much attention to evaluating potential mediators of change that could properly explain their results and clarify why and how the combined interventions did or did not work. Moreover, only three studies included any empirical (or other) test for mediators of change in order to explain how the combined intervention worked. These studies suggest that enhancing employees’ sense of job control (i.e., decision authority over their jobs), social support (e.g., positive feedback from supervisors), participation in decision-making (e.g., selecting stressors and mismatches) and reducing workload can facilitate rehabilitation among employees who are currently either working or absent on sick leave. The results of the three studies involving empirical evaluation of factors mediating change are supported by the fact that all of the studies share specific theoretical (or other) assumptions concerning the importance of involving employees in decision-making, enhancing their job control and social support and reducing stressors (e.g., high workload).

### 4.2. Scientific Implications 

To our knowledge, this systematic review is the first to focus on combined interventions. Although previous reviews have included combined interventions [40,41], the methods underlying their reviews have exhibited many limitations (e.g., using only two or three electronic databases; limiting the search period from 1995 to 2005; and not assessing study quality). The present review addresses these limitations by using seven relevant databases, expanding the search to include all studies since the emergence of the burnout concept (>1970), assessing the risk of bias and including only studies using the MBI or RTW to ensure comparability with regard to the outcome of interest. In addition, two of the “combined interventions” addressed in the review by Awa, Plaumann and Walter [41] were organization-directed (professional supervision [42] and work-shift evaluations [43]) rather than combined interventions and they were thus not included in the present review. Additionally, none of these reviews attempted to identify and describe the mediators of change to explain how combined interventions worked, which was an additional aim of this study. Hence, this systematic review strongly builds upon and complements research on how to effectively facilitate rehabilitation, that is, reducing burnout complaints and promoting a full RTW.

Indicated by the results of this review, the combined interventions did not lead to much improvement in two specific dimensions of burnout: personal accomplishment and professional efficacy. There has long been uncertainty with regard to including these two aspects as dimensions of burnout, as they could be interpreted as either a cause or consequence of burnout [44]. On the one hand, exhaustion might indicate a lack of personal accomplishment (or professional efficacy). On the other hand, a lack of efficacy (or accomplishment) could result from poor performance due to exhaustion [45]. It would therefore be interesting for future studies to provide further clarification on the role of personal accomplishment or professional efficacy as either a cause or consequence of burnout.

With regards to the mediators of change (whether measured directly or merely described), the results are very much in line with central theories on burnout. For example, studies inspired by the Job Demands-Resources Model consistently show that job resources (e.g., job control, social support) are not only negatively related to burnout but also associated with multiple positive outcomes, including enhanced job satisfaction and organizational commitment [16,46]. In a similar vein, a recent cross-sectional study demonstrates that job control and social support are strongly correlated with the ability of employees to participate and be productive in a sustainable and meaningful way [47]. At the same time, the results of this review indicate that reducing workload (i.e., a job demand) can also influence burnout complaints, thus suggesting that interventions should aim to build job resources while addressing stressors. Future intervention studies should continue to clarify the role of job resources and stressors with respect to reducing burnout complaints.

The studies evaluated in this review also reflect the expectation that facilitating partial RTW can predict full RTW. These expectations were not supported by empirical evidence. One possible explanation could be that employees who failed to attain a sustainable RTW toward the end of the follow-up period had more severe burnout complaints and therefore needed a longer period of partial RTW. However, study 10 showed that participants in the combined intervention faster RTW than participants in the control group while their burnout complaints did not improve. This indicates that reducing burnout complaints and facilitating a sustainable RTW cannot be seen as a single phenomenon, which is in line with previous studies [48].

This review assessed studies involving employees from a variety of professions (e.g., healthcare, construction work) and therefore a variety of working contexts. Although the results suggest that combined interventions have beneficial effects on reducing burnout complaints and promoting full RTW for multiple professions, they do not necessarily mean that a given intervention will produce the same effects in a different working context. Similarly, this review also includes a study involving self-employed people, who were thus also business owners. Self-employed individuals differ from employees in several aspects. For example, studies have demonstrated that self-employed people are characterized by strong levels of job control, job insecurity, decision latitude, work demands, intrinsic motivation to work and low levels of social support in their work [49]. Although the effect of the combined intervention aimed at facilitating full RTW among self-employed people (Study 10) was promising, it does not mean that employees (who are not self-employed) on sick leave would automatically benefit from the same combined intervention.

### 4.3. Practical Implications 

The results of this review suggest that combined rehabilitation interventions are effective (at least to a certain extent) in facilitating rehabilitation among employees who are currently working or absent on sick leave. In general, all of the studies share common theoretical (or other) assumptions concerning the importance of involving employees in decision-making and enhancing their sense of job control and social support. These assumptions are further supported by the results of three studies that involved the empirical evaluation of such mediators of change with regard to burnout complaints. From the perspective of promoting workplace health, it could be worthwhile to build such resources while addressing job demands (e.g., excessive workload) in order to alleviate burnout complaints. Reflecting on the mediators of change—particularly participation in decision-making and social support—the results suggest that simply paying attention to employees (i.e., listening to them and addressing their needs) is important to the facilitation of rehabilitation. This knowledge could be applied directly in practice.

### 4.4. Limitations 

Although all combined interventions were effective in facilitating rehabilitation, nine studies had a high risk of bias and one study was assessed as having a moderate risk of bias. This substantial risk of bias has a direct impact on the robustness of the findings of this review. More specifically, it is unclear whether the effects presented in a given study were due to the combined intervention or to the study design as such. The results of this review should therefore be interpreted with caution. It should be emphasized that the risk of bias assessment does not judge the “quality of the included studies,” rather, it aims to identify possible bias based on the context in which the studies were conducted, thereby assessing the “quality of the evidence.” Future studies are challenged to enhance the robustness of the study designs, while taking into account the complexity of the combined interventions, which is a common challenge in (workplace) health promotion research [50].

It should also be noted that the interventions addressed in this review were only general described in terms of content and specific theoretical (or other) assumptions. Future studies should therefore provide more in-depth information on the theories or assumptions on which the interventions are built, as well as on how (and by whom) the interventions were developed, implemented and evaluated, in addition to providing clear definitions for mediators of change. In addition to the quantitative measurement and assessment of mediators of change with regard to their effects on the outcome variables, it would be interesting to evaluate such mediators in qualitative terms. For example, the quantitative measurement and analysis of participation in decision-making does not say anything about how, why and under which circumstances employees were involved. In addition to evaluating the effect of combined interventions on rehabilitation, therefore, research on burnout could benefit from qualitative process evaluation, which could provide further information on why a combined intervention did or did not work. Finally, the duration and intensity of the combined interventions differed substantially, ranging from three sessions of three hours each to six monthly sessions of four hours each. It is therefore impossible to determine exactly when a given intervention will work.

This review is subject to several limitations. Publication bias is likely to have influenced our results. Given that studies resulting in negative or no effects are often not published, we were obviously unable to include them in our review. Similarly, because our review includes only articles published in English, it overlooks any relevant studies published in other languages. Also, although the MBI is used as the golden stand for measuring burnout, it is also known to have multiple conceptual, technical and practical issues [45]. For example, it does not measure all burnout symptoms (e.g., depressive feelings and psychosomatic tension complaints), which are often the first reasons why employees seek help. Recently, a new instrument—the Burnout Assessment Tool (BAT)—has been developed to address the issues associated with the MBI [45]. For this reason, we also attempted to retrieve studies that evaluated combined interventions using the BAT by conducting an additional search on October 10, 2019. Unfortunately, this search did not reveal any additional studies. Finally, though the tool used for the risk of bias assessment has been evaluated as strong [28,29], we did not systematically test the instrument for failure in similar studies and we do not have evidence to state how and where the tool breaks down; that is, we cannot be certain on its validity and reliability.

## 5. Conclusions

All nine combined (both person-directed and organization-directed) interventions showed a positive effect on facilitating rehabilitation among employees who are currently working or on sick leave due to burnout. Although the risk of bias of the included studies is considerably high, the results show that the mediators of change addressed—job control, social support, participation in decision-making and workload—contribute to employees’ rehabilitation. Further studies are challenged to enhance the robustness of the study designs while incorporating the complexity of combined interventions, preferably by adding qualitative process evaluations besides measuring effects. In addition, research on burnout interventions could benefit from qualitative process evaluations aimed at unravelling how and why interventions do or do not work. Finally, with caution, workplace health promotion practitioners are encouraged to facilitate rehabilitation by building job resources while eliminating stressors in the workplace.

## Figures and Tables

**Table 1:** Included articles (N = 10).

Author/s, Country	Setting, Design	Study Aim, Outcome/s	Participants	Controls	Theoretical Framework	Interventions	Mediators of Change Measured	Duration	Pre-Test (T1), Post-Test (T2), Follow-Up (T3)	Results	Risk of Bias
**White-collar workers**											
Studies 1,2 [30,31]; Sweden	Employees on sick leave due to burnout; identified from a social insurance register; controlled clinical trial design	Promoting RTW; RTW (sick leave percentage)	Workers with burnout; confirmed by medical examination and questionnaire interview (n = 74)	Workers with burnout who were not interested in participating in the intervention; no intervention (n = 74)	Job-person (mis)match	Combined intervention: a convergence dialogue meeting (i.e., dialogue between the patient and the supervisor to find solutions to facilitate RTW)	Partial work resumption expected to foster full RTW	Half-day seminar, 1.5-hour meeting	After 18 and 30 months, the total sick leave↓ in the combined intervention group, as compared to the control group	After 18 and 30 months, the total sick leave↓ in the combined intervention group, as compared to the control group	High
Study 3 [32]; Netherlands	Staff members of 29 oncology wards of 18 general hospitals; quasi-experimental design	Reducing burnout complaints; MBI-HSS (EE, DP)	Staff members at risk of developing burnout; randomly selected from 9 wards (n = 260)	Staff members; remaining 19 wards; no intervention (n = 404)	Not reported	Combined intervention: a staff support group and a participatory approach (n = 260)	Job control, social support, participation in decision-making, quantitative demands and patient-related emotional demands	6 monthly sessions of 4 hours each	T1–before the intervention T2–6 months later, directly after the intervention ended T3–6 months after the intervention ended	In the combined intervention, EE↓ at both T2 and T3, DP↓ at T3 compared to the control group	High
Study 4 [33]; Hong Kong	Construction-related professionals engaged in property development, consulting and contracting companies; quasi-experimental design	Reducing burnout complaints; MBI-GS (EE, CY, PE)	Workers at risk of developing burnout; all workers worked in the same company (n = 55)	None	Job-person (mis)match	Combined intervention; based on job-redesign addressing stressors and resources in the workplace (n = 55)	None	A period of one year. The frequency of the interventions differed according to the activity	T1–before the intervention T2–1 year after the intervention T3–none	EE↓, CY↓ after the combined intervention; PE↔	High
Study 5 [34]; Finland	White-collar women diagnosed as having various job-related psychological health problems (e.g., burnout); quasi-experimental design	Reducing burnout complaints; MBI-GS (EX, CY, PE scores)	Female white-collar workers; diagnosed by physicians based on their medical report application (n = 20 + 32)	Female white-collar workers; awaiting treatment (n = 12).	Based on job-person (mis)match	Traditional intervention: primary focus on the individual but when necessary, also on the individual-organizational interface (n = 32) Combined intervention: similar to the traditional intervention but based on a participatory approach (n = 21)	Job control, social support, participation in decision-making	One year with two rehabilitation periods (12 and 5 days, respectively)	T1–before the intervention T2–after the first part of the intervention, 4 months after T1 T3–after the second part of the intervention, 8 months after T2	In the combined intervention, EX↓ between T1 and T2 and between T1 and T3; CY↓ between T1 and T2; PE↔ In the traditional intervention, EX↔, CY↔, DP↔ In the control group, CY↓ between T1 and T2; CY↔, DP↔	High
Study 6 [35]; Norway	Staff members working with people with intellectual disabilities in two municipalities; 2 groups, pre-test/post-test design	Reducing burnout complaints; MBI-GS (EX, CY, PE scores)	Staff working in one municipality at risk of developing burnout (n = 79)	Staff working in a different municipality; no intervention (n = 33)	Job-person mismatch	Combined intervention: focusing on the individual (e.g., exercise in a health club) and the organization (e.g., improving the working schedule) (n = 79)	None	A period of 10 months. The frequency of the interventions differed depending on the activity	T1–before the intervention T2–after the intervention, (i.e., after 10 months) T3–none	In the combined intervention EX↓ after the intervention, as compared to the control group; CY↔, PE↔ In the control group, EX↔, CY↔, PE↔	High
**Healthcare workers**											
Study 7 [36]; USA	General surgery residents working at the University of Arizona; one group, pre-test/post-test design	Reducing burnout complaints; MBI-GS (EX, CY, PE)	Staff members at risk of developing burnout; the intervention was part of their formal (on the job) education (n = 49)	None	Not reported	Combined intervention: multiple activities (e.g., mindfulness sessions, team building) (n = 49)	None	A period of one year. Monthly, interactive sessions were provided	T1–before the intervention T2–One year after the implementation of the intervention T3–none	EE↓ after the combined intervention; CY↔, PE↔	High
Study 8 [37]; England	Staff working in an in-patient alcohol ward; one group, pre-test/post-test design	Reducing burnout complaints; MBI (EE, DP, PA)	Staff members at risk of developing burnout; all staff were invited to participate in the intervention (n = 19)	None	Demand-Control Support Job Stress Model	Combined intervention: managing stress at the individual, team and organizational level and on understanding the causes and consequences of aggression (n = 19)	None	Two-day training with two weeks between the training days	T1–3 months before the intervention T2–1 month after the intervention ended T3–none	PA↑after the combined intervention, EE↔, DP↔	High
Study 9 [38]; USA	Staff representing 15 departments (e.g., nursing, pharmacy, housekeeping); one group, retrospective pre-test/post-test design	Reducing burnout complaints; MBI-HSS (EE, CY, PA)	Staff members at risk of developing burnout; a stratified random sample reflecting all departments (n = 51)	None	Not reported	Combined intervention: based on experiential techniques (e.g., team building and enhancing self-esteem) (n = 51)	None	Three sessions of three hours each	T1–3 months before the intervention T2–1 month after the intervention ended T3–none	EE↓, PA↑ after the combined intervention; CY↔	High
**Self-employed**											
Study 10 [39]; Netherlands	Self-employed individuals on sick leave due to work-related psychological complaints (e.g., burnout); controlled clinical trial design	Reducing burnout complaints, promoting RTW; MBI-NL (EX, DP, PE scores), RTW (mean number of days to partial and full return to work)	Self-employed; screened by psychologists (n = 40 + 40)	Self-employed; asked to postpone their treatment for four months (n = 42)	Not reported	Person-directed intervention: CBT; focused on cognitive restructuring (n = 40) Combined intervention: CBT-based stress management and meetings with labor experts aimed at changing the work context (n = 40)	None	11 bi-weekly sessions of approximately 45 minutes per session 5 to 6 sessions of approximately 1 hour, twice per week	T1-before the intervention T2-4 months after the onset of the intervention T3-10 months after the onset of the intervention	EE↓, DP↓; PE↔, regardless of the intervention Shorter time to partial and full RTW for participants in the combined intervention, as compared to those in the person-directed intervention and control group	Moderate
↑ = significant increase; ↔ = no significant change; ↓ = significant decrease											

Abbreviations: CCT = Controlled Clinical Trial; MBI = Maslach Burnout Inventory; MBI–GS = Maslach Burnout Inventory–General Survey; MBI–HSS = Maslach Burnout Inventory–Human Services Survey; MBI–NL = Maslach Burnout Inventory–Netherlands; RTW = Return to Work; EX = Exhaustion; CY = Cynicism; DP = Depersonalization; PA = Personal Accomplishment; PE = Professional Efficacy.

## Supplementary Materials

The following are available online at https://www.mdpi.com/1660-4601/17/1/55/s1, Table S1: The PRISMA Checklist; Table S2: The Assessment of Bias Scores of the Included Studies.
